# Reduced mechanical function of the left atrial predicts adverse outcome in pregnant women with clustering of metabolic risk factors

**DOI:** 10.1186/s12872-021-02082-7

**Published:** 2021-05-29

**Authors:** Xiaoguang Ye, Zhitian Li, Yidan Li, Qizhe Cai, Lanlan Sun, Weiwei Zhu, Xueyan Ding, Dichen Guo, Yunyun Qin, Xiuzhang Lu

**Affiliations:** 1 Department of Echocardiography, Heart Center, Beijing ChaoYang Hospital, Capital Medical University, 8 Gongren Tiyuchang Nanlu, Chaoyang District, Beijing, 100020 China; 2 Department of Thoracic Surgery, Beijing ShiJiTan Hospital, Capital Medical University, Beijing, China

**Keywords:** Echocardiography, Metabolic risk factors, Left atrial function

## Abstract

**Introduction:**

The left atrial (LA) strain and strain rate are sensitive indicators of LA function. However, they are not widely used for the evaluation of pregnant women with metabolic diseases. The aim of this study was to assess the LA strain and strain rate of pregnant women with clustering of metabolic risk factors and to explore its prognostic effect on adverse pregnancy outcomes.

**Materials and methods:**

Sixty-three pregnant women with a clustering of metabolic risk factors (CMR group), fifty-seven women with pregnancy-induced hypertension (PIH group), fifty-seven women with gestational diabetes mellitus (GDM group), and fifty matched healthy pregnant women (control group) were retrospectively evaluated. LA function was evaluated with two-dimensional speckle-tracking imaging. Iatrogenic preterm delivery caused by severe preeclampsia, placental abruption, and fetal distress was regarded as the primary adverse outcome.

**Results:**

The CMR group showed the lowest LA strain during reservoir phase (LASr), strain during contraction phase (LASct) and peak strain rate during conduit phase (pLASRcd) among the three groups (*P* < 0.05). LA strain during conduit phase (LAScd) and peak strain rate during reservoir phase (pLASRr) in the CMR group were lower than those in the control and GDM groups (*P* < 0.05). Multivariable Cox regression analysis demonstrated systolic blood pressure (HR = 1.03, 95% CI 1.01–1.05, p = 0.001) and LASr (HR = 0.86, 95% CI 0.80–0.92, p < 0.0001) to be independent predictors of iatrogenic preterm delivery. An LASr cutoff value ≤ 38.35% predicted the occurrence of iatrogenic preterm delivery.

**Conclusions:**

LA mechanical function in pregnant women with metabolic aggregation is deteriorated. An LASr value of 38.35% or less may indicate the occurrence of adverse pregnancy outcomes.

## Introduction

Pregnancy is a special physiological period for women and is accompanied by significant changes in the cardiovascular system and metabolism. During normal pregnancy, these physiological changes are beneficial for women going through this special time and ensure the healthy growth and development of the fetus. However, pregnant women who exhibit metabolic abnormalities usually have a greater risk of cardiovascular events [1]. Indeed, pregnant women with pregnancy-induced hypertension (PIH) or gestational diabetes mellitus (GDM) have a higher risk of cardiovascular disease (CVD) during pregnancy and postpartum [2, 3]. A previous study showed that there may be a common pathophysiological basis for multiple metabolic abnormalities in pregnant women [4]. For example, insulin resistance and hyperinsulinemia may be common characteristics of women with PIH or GDM, which are closely related to a high body mass index (BMI) before pregnancy [5, 6]. Common metabolic risk factors during pregnancy include pre-pregnancy overweight/obesity, pregnancy dyslipidemia, hyperglycemia and high blood pressure. Previous studies focused more on the correlation between a single metabolic disease and cardiac function than on the combined effects of multiple metabolic diseases/risk factors and cardiac function, which is very common in pregnant women.

Previous studies have shown that metabolic diseases during pregnancy impair left ventricular (LV) function, especially LV diastolic function, in pregnant women [7−9]. Left atrial (LA) function is a sensitive indicator of cardiac diastolic function, although a small number of studies have focused on left atrial function in pregnant women with single metabolic abnormality [10], it is not known whether the left atrial function of pregnant women with multiple metabolic abnormalities is worse. In addition, it is not known whether abnormal left atrial function can predict poor pregnancy outcome.

Two-dimensional speckle-tracking imaging (2D-STI) can accurately measure LA function and allows for direct and angle-independent analysis of myocardial deformation. A previous study successfully used 2D-STI to evaluate the changes in LA function in women with normal pregnancies [11].

In this study, our aim is to compare whether there are differences in left atrial function between pregnant women with multiple metabolic abnormalities and those with single metabolic abnormalities (gestational hypertension and gestational diabetes) by 2D-STI, and to examine the relationship between left atrial dysfunction and the risk of adverse pregnancy outcome.

## Materials and methods

### Study population

A retrospective observational study was performed using medical records of singleton pregnancy patients in Beijing Chao-Yang Hospital, Capital Medical University from 2017 to 2020. The patient population consisted of 227 women (mean age 31.9 years, range 24 to 41 years). The subjects were consecutive patients. All pregnant women completed all normal antenatal examinations. According to the following diagnostic criteria, we divided the participants into four groups: metabolic risk factors (CMR) group, PIH group, GDM group, and control group. PIH was diagnosed as blood pressure ≥ 140/90 mmHg measured on two separate occasions after the 20th week of gestation. GDM was defined according to the International Association of Diabetes and Pregnancy Study Groups (IADPSG) criteria [12] when any of the following criteria were met during a 75-g oral glucose tolerance test (OGTT) between 24 and 28 gestational weeks: 1) fasting plasma glucose (FPG) ≥ 5.1 mmol/L; 2) 1-h plasma glucose (1hPG) during OGTT ≥ 10.0 mmol/L; and 3) 2hPG during OGTT ≥ 8.5 mmol/L. The pre-pregnancy body mass index (pre-BMI) was calculated as weight (kg)/height (m)^2^ and a pre-BMI ≥ 25 kg/m^2^ was considered to be overweight according to the diagnostic criteria of the World Health Organization. Maternal venous blood samples were drawn in the morning after overnight fasting for ≥ 8 h to measure maternal plasma triglyceride (TG) and high-density lipoproteins–cholesterol (HDL-C) before the 20th week of gestation. The CMR group consisted of pregnant women with three or more of the following risk factors: pre-BMI ≥ 25 kg/m^2^; PIH; GDM; TG ≥ 3.49 mmol/L; HDL-C < 1.3 mmol/L. The PIH group consisted of pregnant women with PIH but without other metabolic risk factors. The GDM group consisted of pregnant women with GDM but without other metabolic risk factors. The control group consisted of age- and gestational week-matched healthy pregnant women without any metabolic risk factors. Any pregnant women who had one of the following were excluded from this study: congenital heart disease; hypertension, diabetes or other chronic diseases before pregnancy; fetal malformation; placental abnormality; smoking, drinking, or drug use; Serious obstetrical complications such as preeclampsia and fetal distress have occurred when pregnant women underwent echocardiography.

The study approved by the Beijing Chaoyang hospital ethics committee, which waived the need for informed consent in compliance with China law on retrospective studies of anonymized data and was conducted in compliance with the Declaration of Helsinki.

### Echocardiography

Transthoracic echocardiography as a routine examination in the second trimester of pregnancy was performed for all participants as recommended by the American Society of Echocardiography and the European Association of Cardiovascular Imaging [13, 14]. The results of echocardiography were reported to the obstetrician and gynecologist. Images were obtained with the patient in the left lateral decubitus position using a commercially available ultrasound machine (EPIQ 7C, Philips Healthcare, MA, USA) equipped with an X5-1 multiphase-array probe. Tissue Doppler imaging (TDI) was performed as well as color, 2D, pulsed- and continuous-wave Doppler imaging according to the standard protocols. LA images were obtained in the apical four- and two-chamber views at high frame rates (> 60 frames/sec) and three consecutive cardiac cycles were recorded. Interventricular septum thickness (IVSd) and posterior wall thickness (PWd)were obtained in the parasternal long-axis view. End-systolic volume (LVESV), LV end-diastolic volume (LV EDV), LV ejection fraction (LV EF), stroke volume (SV), and LA volume (LAV) were obtained using the biplane modified Simpson’s method. The LAV index (LAVi) was calculated based on the body surface area (BSA). Other calculated indicators included the TDI mitral valve E/e' ratio, mitral valve E/A ratio, and LA ejection fraction (LA EF). The apical four-, three-, and two-chamber views were used to obtain the LV global longitudinal systolic strain (LV GLS).

### Analysis of LA strain and strain rate

LA strain was obtained by 2D-STI. Three stable consecutive cardiac cycles were recorded and stored for offline analysis using QLAB 10.8 software (Philips Healthcare). The region of interest was selected by using the point-and-click method to demarcate the LA wall, and a 12-segment model was employed to generate longitudinal strain and strain rate curves. After setting zero strain at the R wave (LVED) together with R-R gating, the LA strain pattern was characterized by a predominant positive wave that peaked at the end of the ventricular systole, followed by two distinct descending phases in the early and late diastoles. The LA parameters were measured as follows: reservoir function, which was appraised based on the strain during reservoir phase (LASr) and peak strain rate during reservoir phase (pLASRr); conduit function, which was appraised based on the conduit strain (LAScd) and peak strain rate during conduit phase (pLASRcd); and booster pump function, which was appraised based on the strain during contraction phase (LASct) and peak strain rate during contraction phase (pLASRct).

### Adverse outcome

The primary adverse outcome in this study was iatrogenic preterm delivery, which was defined as a prenatal cesarean section for medical reasons between 28 and 36^6/7^ weeks of pregnancy, with no premature rupture of membranes and spontaneous preterm delivery [15]. The causes of iatrogenic preterm delivery included severe preeclampsia, placental abruption, and/or fetal distress, which were defined as adverse pregnancy outcomes in this study.

### Statistical analysis

Statistical analysis was performed using SPSS version 23.0 software (IBM SPSS Statistics, v23). Quantitative data were expressed as the mean ± standard deviation (SD), median, or interquartile range. Categorical data were expressed as the number of patients (percentage) whenever appropriate. Normal distribution of data was evaluated with the Kolmogorov–Smirnov test. One-way analysis of variance followed by Bonferroni correction and the Kruskal–Wallis H test was used for the comparison of normally distributed quantitative data, and the χ2 test was used for categorical data. A P-value less than 0.05 was defined as statistical significance.

Receiver operating characteristic (ROC) curves and the area under the curve (AUC) was used to quantify the global performance of LA strain in determining the occurrence of iatrogenic preterm delivery. Using the discriminatory cut-off, we divided the cohort into two groups; greater than the cut-off value and less than or equal to the cut-off value. The survival curve for gestation at delivery was used to evaluate whether there was a difference between the layers of LASr. The logarithmic rank test was used to compare the survival curves for the two groups. Univariable cox regression analysis was used to evaluate the association between clinical and echocardiographic parameters for the endpoint. Variables with a p value of < 0.05 were considered for inclusion in the multivariable Cox proportional hazards models to identify the independent predictors of the study endpoints. Inter-and intra-observer variability was evaluated using intraclass correlation coefficients (ICCs).

## Results

### Demographic and clinical characteristics of study participants

A total of 227 pregnant women were enrolled in this study and divided into the following four groups based on their clinical characteristics: CMR group (n = 63), PIH group (n = 57), GDM group (n = 57), and control group (n = 50). The demographic and major clinical characteristics, including pre-BMI, gestational weight gain, BP, TG, HDL-C, PFG, and adverse pregnancy outcomes are presented in Table 1. There were no significant differences with regard to age or gestational weeks in the echocardiography results among these groups. The CMR group had a significantly higher BSA and iatrogenic preterm delivery ratio than the GDM and control groups (*P* < 0.05). As expected, pre-BMI, TG, and HDL-C values were significantly higher in CMR patients than in the other three groups (*P* < 0.05). The CMR group had a significantly higher SBP and diastolic blood pressure (DBP) than the GDM and control groups (*P* < 0.05). Furthermore, FPG was significantly higher (*P* < 0.05) in CMR pregnant women than in PIH and control pregnant women. There was a significant difference in gestational weight gain among these groups (*P* < 0.05), but no significant difference was seen after Bonferroni correction.

**Table 1:** Demographic and clinical characteristics of participants

Clinical parameters	Control (n = 50)	GDM (n = 57)	PIH (n = 57)	CMR (n = 63)	*P* value
Age, years	32.06 ± 3.98	32.16 ± 4.14	31.39 ± 3.45	32.24 ± 4.13	0.659
Gestational age at echocardiography, weeks	22.10 ± 1.54	21.63 ± 1.81	22.28 ± 1.89	22.30 ± 2.41	0.228
BSA, m^2^	1.73 ± 0.11	1.78 ± 0.11	1.79 ± 0.12	1.86 ± 0.15^†,§^	< 0.001
Pre-BMI, Kg/m^2^	22.10 ± 3.02	22.91 ± 3.25	22.06 ± 2.88	26.36 ± 3.60^†,§,‡^	< 0.001
Gestational weight gain, Kg	13.58 ± 4.12	12.17 ± 5.03	14.74 ± 5.07	12.36 ± 5.90	0.026
TG, mmol/L	2.85 (2.28, 3.65)	2.87 (2.11, 3.74)	3.23 (2.59, 4.44)	4.61 ± 1.30^†,§,‡^	< 0.001
HDL-C, mmol/L	2.03 ± 0.40	1.97 ± 0.41	1.83 ± 0.43	1.58 ± 0.46^†,§,‡^	< 0.001
Systolic blood pressure, mmHg	118.40 ± 9.11	118.72 ± 7.64	152.74 ± 9.89	151.52 ± 10.64^†,§^	< 0.001
Diastolic blood pressure, mmHg	73.46 ± 7.22	74.19 ± 5.40	93.35 ± 10.09	93.84 ± 8.58^†,§^	< 0.001
FPG, mmol/L	4.26 ± 0.46	5.13 ± 0.88	4.25 ± 0.45	5.02 ± 0.83^†,‡^	< 0.001
Weeks at delivery, weeks	39.16 ± 1.13	38.63 ± 1.58	37.72 ± 2.90	37.17 ± 1.91^†,§^	< 0.001
Iatrogenic preterm delivery, Pts (%)	0/50 (0%)	3/57 (5.26%)	12/57 (21.05%)	27/63 (42.86%)^†,§^	< 0.001

Values are the mean ± standard deviation, median (interquartile range), or n (%)

BSA, Body Surface Area; pre-BMI, pre-pregnancy body mass index; TG, triglyceride; HDL-C, high-density lipoproteins-cholesterol; FPG, fasting plasma glucose. †P < 0.05, CMR group vs Control group. §P < 0.05, CMR group vs GDM group. ‡P < 0.05, CMR group vs PIH group

### Conventional echocardiographic parameters

We compared the main traditional echocardiographic parameters among these groups (Table 2). The CMR group had greater LVEDV and LVESV than the control group (*P* < 0.05), but showed no significant difference compared with the PIH and GDM groups. The CMR group also had a normal SV but lower LVEF than the control group (*P* < 0.05). In addition, CMR patients had a higher LV filling pressure as indicated by a larger E/e’ ratio as well as greater LAVi compared with the control group (*P* < 0.05). Furthermore, the CMR group had thicker IVSd and PWd and a lower mitral valve E/A ratio than the control and GDM groups (*P* < 0.05), but no differences in these parameters were observed between the CMR and PIH groups.

**Table 2:** Conventional echocardiographic parameters for participants

Variables	Control (n = 50)	GDM (n = 57)	PIH (n = 57)	CMR (n = 63)	*P* value
LVEDV, mL	85.11 ± 15.74	91.34 ± 15.84	89.04 ± 17.39	93.97 ± 16.02^†^	0.034
LVESV, mL	30.09 ± 7.02	32.87 ± 7.13	32.46 ± 6.9	35.47 ± 7.48^†^	0.001
SV, mL	55.02 ± 10.25	58.47 ± 10.39	56.57 ± 12.06	58.5 ± 10.13	0.266
LV EF, % (biplane Simpson)	64.77 ± 4.38	64.04 ± 4.12	63.42 ± 3.92	62.45 ± 3.87^†^	0.021
IVSd, cm	9.17 ± 0.75	9.03 ± 0.68	10.18 ± 1.23	10.1 ± 1.25^†,‡^	< 0.001
PWd, cm	8.96 ± 0.6	9.05 ± 0.65	9.91 ± 1.23	9.92 ± 0.99^†,§^	< 0.001
Mitral E/A ratio	1.28 ± 0.33	1.27 ± 0.40	1.09 ± 0.27	1.03 ± 0.27^†,§^	< 0.001
LV E/e’	6.83 ± 1.64	8.19 ± 2.34	8.58 ± 2.74	9.07 ± 2.76^†^	< 0.001
LV GLS (%)	23.59 ± 2.26	22.09 ± 2.25	20.89 ± 2.14	20.33 ± 2.19^†,§^	< 0.001
LAVi, ml/m2	20.99 ± 4.61	22.08 ± 4.95	23.02 ± 5.26	23.69 ± 6.18^†^	0.048
LA EF, % (biplane Simpson)	73.77 ± 5.52	71.59 ± 5.84	70.98 ± 7.23	65.96 ± 6.59^†^	0.008

Values are the mean ± standard deviation, median (interquartile range)

LVEDV, left ventricular end-diastolic volume; LVESV, left ventricular end-systolic volume; SV, Stroke volume; LV EF, left ventricular ejection fraction; IVSd, interventricular septum thickness; PWd, posterior wall thickness; LV E/e’, ratio of early diastolic mitral flow velocity to early diastolic peak velocity of lateral mitral annulus; LV GLS, left ventricular global longitudinal strain; LAVi, left atrial volume index; LA EF, left atrial ejection fraction. ^†^*P* < 0.05, CMR group vs Control group. ^§^*P* < 0.05, CMR group vs GDM group. ^‡^*P* < 0.05, CMR group vs PIH group

### LA strain and strain rate

The comparison of the LA strain and strain rate among the study groups is shown in Table 3. Compared with three other groups, patients with CMR showed the lowest LASr (*P* < 0.001 versus control; *P* = 0.014 versus PIH; *P* < 0.001 versus GDM), LASct(*P* = 0.001 versus control; *P* = 0.025 versus PIH; *P* = 0.002 versus GDM) and pLASRcd (*P* < 0.001 versus control; *P* = 0.004 versus PIH; *P* < 0.001 versus GDM). LAScd(*P* < 0.001 versus control; *P* = 0.008 versus GDM) and pLASRr(*P* < 0.001 versus control; *P* < 0.001 versus GDM) in the CMR group were lower than those in the control and GDM groups, but there were no differences between the CMR and PIH groups. Notably, pLASRct was comparable among CMR, PIH and GDM groups.

**Table 3:** Left atrial functional parameters assessed based on 2D-STI of participants

Variables	Control (n = 50)	GDM (n = 57)	PIH (n = 57)	CMR (n = 63)	*P* value	*P* value^*^	*P* value^**^	*P* value^***^
LASr (%)	48.63 ± 6.11	44.38 ± 5.77	40.27 ± 5.94	37.10 ± 4.67	< 0.001	< 0.001	< 0.001	0.014
pLASRr (sec^−1^)	2.82 ± 0.54	2.67 ± 0.46	2.38 ± 0.54	2.22 ± 0.52	< 0.001	< 0.001	< 0.001	0.632
LAScd (%)	29.86 ± 5.87	26.68 ± 6.14	23.72 ± 6.70	23.00 ± 6.11	< 0.001	< 0.001	0.008	1.000
pLASRcd (sec^−1^)	3.33 ± 0.60	3.15 ± 0.65	2.77 ± 0.59	2.36 ± 0.75	< 0.001	< 0.001	0.004	< 0.001
LASct (%)	18.75 ± 6.29	17.7 ± 6.68	16.72 ± 6.84	14.10 ± 5.64	0.001	0.001	0.002	0.025
pLASRct (sec^−1^)	3.42 ± 0.8	3.22 ± 0.62	3.07 ± 0.64	3.05 ± 0.67	0.019	0.033	1.000	1.000

Values are the mean ± standard deviation

LASr, strain during reservoir phase; pLASRr, peak strain rate during reservoir phase; LAScd, strain during conduit phase; pLASRcd, peak strain rate during conduit phase; LASct, strain during contraction phase; pLASRct, peak strain rate during contraction phase. * P, CMR vs. control; ** P, CMR vs. GDM group;*** P, CMR vs. PIH

### Determination of predictors of adverse outcome

In this study, a total of 42 patients (18.50%) had iatrogenic preterm delivery. Among them, 27 (42.86%) were in the CMR group, 12 (21.05%) were in the PIH group, and three (5.26%) were in the GDM group (Table 1). According to the outcome event, pregnant women with metabolic diseases were divided into two groups. Their clinical and ultrasonic parameters are shown in Table 4.

**Table 4:** Clinical and echocardiographic parameters of pregnant women with metabolic diseases comparing with and without adverse outcome (*N* = 177)

Variables	Women with adverse outcome (n = 42)	Women without adverse outcome (n = 135)	*P* value
Age, years	32.02 ± 3.76	31.91 ± 3.99	0.871
BSA, m^2^	1.81 ± 0.15	1.81 ± 0.13	1.000
Pre-BMI, Kg/m^2^	25.43 ± 4.19	23.38 ± 3.49	0.002
TG, mmol/L	4.21 ± 1.41	3.83 ± 1.72	0.185
HDL-C, mmol/L	1.57 ± 0.41	1.85 ± 0.46	< 0.001
SBP, mmHg	152.09 ± 13.71	138.00 ± 18.28	< 0.001
DBP, mmHg	96.26 ± 10.24	84.59 ± 11.53	< 0.001
FPG, mmol/L	4.61 ± 0.83	4.87 ± 0.84	0.079
LV EF, %	62.46 ± 3.38	63.50 ± 4.16	0.143
LV E/e’	9.46 ± 2.19	8.41 ± 2.70	0.012
LV GLS (%)	20.36 ± 2.14	21.31 ± 2.31	0.019
LAVi, ml/m^2^	24.87 ± 6.82	22.36 ± 4.94	0.032
LA EF, %	37.69 ± 9.79	40.15 ± 9.35	0.145
LASr (%)	36.05 ± 4.77	41.84 ± 5.97	< 0.001

Values are the mean ± standard deviation

**Fig. 1:**
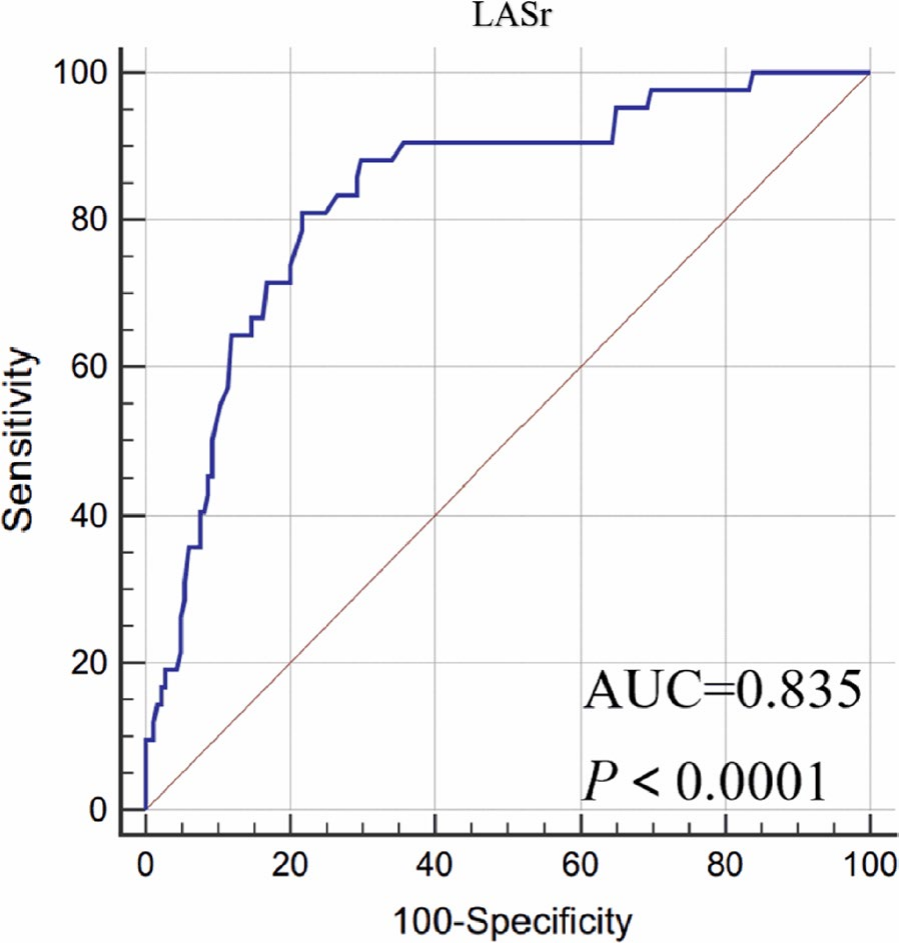
Receiver operating characteristics curve for determination of optimal LASr value in predicting the adverse outcome. A LASr value of 38.35% or less was identified as the cutoff value. AUC, area under the curve; LASr, strain during reservoir phase

**Fig. 2:**
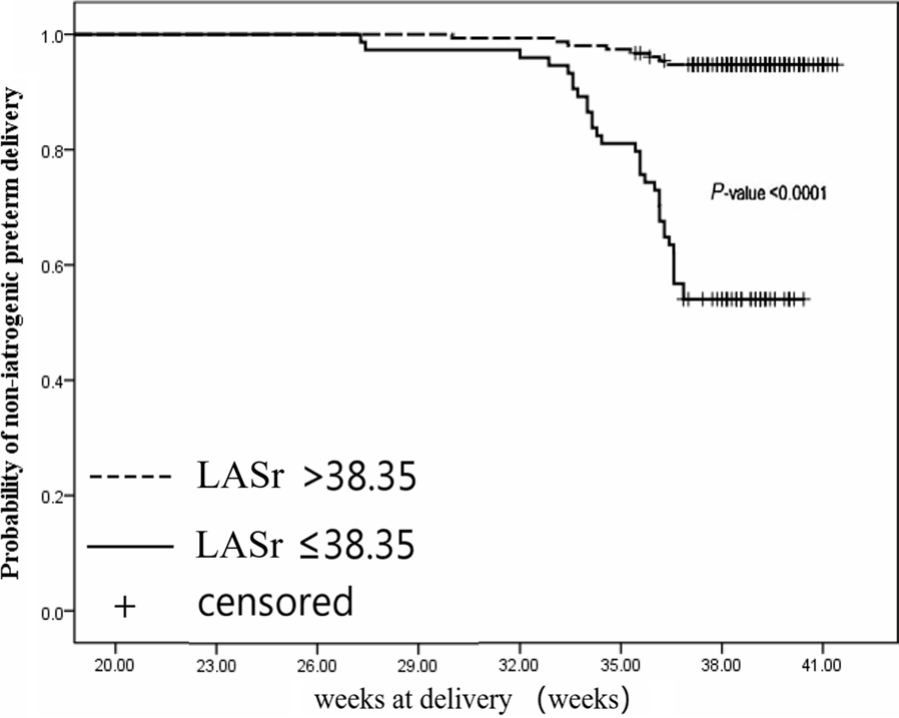
Kaplan–Meyer survival curves for the two ranges of the LASr value in all the subjects. LASr value of 38.35% or less was associated with significantly increased incidence of adverse pregnancy outcome in all subjects. Dotted line represents adverse pregnancy outcome for patients with LASr more than 38.35%. Straight line represents adverse pregnancy outcome for patients with LASr of 23.5% or less. LASr, strain during reservoir phas

BSA, Body Surface Area; pre-BMI, pre-pregnancy body mass index; TG, triglyceride; HDL-C, high-density lipoproteins-cholesterol; SBP, systolic blood pressure; DBP, systolic blood pressure;FPG, fasting plasma glucose; LV EF, left ventricular ejection fraction; LV E/e’, ratio of early diastolic mitral flow velocity to early diastolic peak velocity of lateral mitral annulus; LV GLS, left ventricular global longitudinal strain; LAVi, left atrial volume index; LA EF, left atrial ejection fraction; LASr, left atrial strain during reservoir phase;

On ROC curve analysis, an LASr value ≤ 38.35% predicted iatrogenic preterm delivery with a sensitivity of 78%, a specificity of 81%, and an AUC of 0.835 (Fig. 1). The Kaplan–Meier survival curves for the two strata of the LASr value (> vs. ≤ 38.35%) are shown in Fig. 2. Log-Rank test for the differences between the two curves showed a significant difference (P < 0.0001).

Multivariable Cox regression analysis was performed using variables which showed a significant association to the primary endpoint. This showed SBP and LASr to be independent predictors of iatrogenic preterm delivery with HRs (95% confidence interval [CI]) of 1.03 (1.01–1.05) and 0.86 (0.8–0.92), respectively (Table 5).

**Table 5:** Univariable and multivariable Cox proportional hazard ratio models

Variables	Univariable Cox regression model			Multivariable Cox regression model		
	HR	95% CI	*P* Value	HR	95% CI	*P* Value
SBP	1.05	1.03–1.07	< 0.0001	1.03	1.01–1.05	0.001
FPG	0.85	0.57–1.27	0.42			
TG	1.20	1.03–1.40	0.019	1.04	0.85–1.28	0.706
P-BMI	1.16	1.07–1.26	< 0.0001	1.07	0.99–1.16	0.100
LV EF	0.94	0.87–1.02	0.12			
LV GLS	0.78	0.68–0.90	< 0.0001	0.97	0.85–1.12	0.674
LV E/e’	1.27	0.59–2.74	0.54			
LASr (%)	0.82	0.77–0.87	< 0.0001	0.86	0.80–0.92	< 0.0001

SBP, systolic blood pressure; FPG, fasting plasma glucose; TG, triglyceride; pre-BMI, pre-pregnancy body mass index; LV EF, left ventricular ejection fraction; LV GLS, left ventricular global longitudinal strain; LV E/e’, ratio of early diastolic mitral flow velocity to early diastolic peak velocity of lateral mitral annulus; LASr, left atrial strain during reservoir phase;

### Inter- and intra-observer variability

The inter- and intra-observer variability of LASr was represented by the ICCs and coefficients of variation among participants randomly selected from each group (n = 10). The ICCs for inter- and intra-observer variability were 0.86 and 0.88, respectively, indicating the reliability and reproducibility of our observations in this study.

## Discussion

In this study, we found that pregnant women with multiple metabolic risk factors had more severe reduced LA function than those with PIH or GDM. The LASr cutoff value, below which the risk of iatrogenic preterm delivery (due to severe preeclampsia, placental abruption, and fetal distress) was significantly increased, was 38.35%.

In normal pregnancy, the left atrium is remodeled to meet special hemodynamic and metabolic needs. It is well known that the total blood volume, plasma volume, and red blood cell mass increase significantly during pregnancy, resulting in the expansion of the LA volume. This is supported by findings that the LAVi for normal pregnant women in the second and third trimester is significantly higher than that for normal non-pregnant women [16, 17]. Song et al. [11] demonstrated that the reservoir function and the booster pump function were significantly increased while the conduit function was decreased in normal pregnant women as compared with non-pregnant women. This is a normal physiological adaptation of the LA function to the changes in volume load during pregnancy. However, in pregnant patients with metabolic diseases, the maternal cardiac diastolic function is decreased. In this study, we found that pregnant women with abnormal glucose tolerance had significant differences in the Mitral A wave and TDI mitral E’/A’ ratio compared with control group, which may be explained by the effect of insulin resistance on deteriorating cardiac diastolic function [18]. Melchiorre et al. reported that pregnant women with preterm delivery due to preeclampsia had LV diastolic dysfunction and LA remodeling and these damages remained for one year after delivery [19]. LA function itself is an important indicator of cardiac diastolic function. However, few studies have been performed to study LV function in pregnant women with metabolic diseases. A previous study examined the LA function in pregnant patients with PIH using 2D-STI and reported that the global LA peak strain decreased in these patients, which was associated with postpartum persistent hypertension [10]. Consistent with their findings, we also found in this study that the LA reservoir function, expressed by the LASr value, decreased in the PIH group, furthermore the CMR group had the lowest LASr.

The LA function includes reservoir function, conduit function, and booster pump function. LASr and pLASRr, which appear during the systolic period, represent the reservoir function. LAScd and pLASRcd represent the shortening deformation and rate of atrial myocardium during the early LV diastolic period as well as the function of the LA conduit. LASct and pLASRct are the shortening rates of atrial myocardium during active LA contraction in the late LV diastolic period, representing the function of the booster pump. We found that the CMR group had decreased function in the LA reservoir, conduit, and pump compared to the control and GDM groups. However, compared to the PIH group, only the reservoir function was decreased in the CMR group. Nevertheless, the LASr has been reported to be the most valuable indicator of LV function, which is closely related to the prognosis of heart disease [20−22].

During pregnancy, the physiological enlargement of the breast increases the difficulty of obtaining LV apical images due to the fact that the left ventricle is located in the near field. The left atrium is a mirror of LV systolic and diastolic function. It connects to the pulmonary veins during systole and supplies the blood to the ventricle during diastole to promote ventricular performance [23]. Therefore, LASr is very important to the overall cardiac performance. As we reported here, LASr sensitively reflected the impaired cardiac function of the CMR patients and exhibited a worthy predictive value in the multivariable cox regression analyses. Our study further showed that impaired LA function (LASr ≤ 38.35%) also predicted the adverse outcome of iatrogenic preterm delivery, which caused by severe preeclampsia, placental abruption, and/or fetal distress. This may be because the decline of LA function represents the degree of systemic damage caused by metabolic diseases, especially the cardiovascular system. Previous studies have showed LA function to be a sensitive and reliable prognostic indicator which can be used as a marker of target organ damage in metabolic diseases [24–25].

The relationship between a single metabolic abnormality, such as PIH or GDM, and poor prognosis, has been reported. For instance, the Hyperglycemia and Adverse Pregnancy Outcome (HAPO) study showed that an increase in blood glucose levels during pregnancy is related to a poor prognosis [26]. Another report showed that hypertriglyceridemia is a predictor of macrosomia in non-obese women [27]. Bakker et al. [28] reported that elevated blood pressure during pregnancy negatively affects fetal growth and development, and increases the risk of preterm delivery. However, pregnant women with multiple metabolic abnormalities have a higher risk of adverse pregnancy outcomes. A prospective study involving 5535 pregnant women showed that the more metabolic risk factors a pregnant woman had, the greater the risk of adverse pregnancy outcomes will be (including preterm delivery, pre-eclampsia, GDM, small/large for gestational age) [29]. The study also found that the odds ratio for a cluster of two factors was 3.32 (95% CI 2.69–4.10) and that for a cluster of three and more factors was 10.40 (95%CI 7.37–14.69) [29]. Consistent with the report, in our study, the CMR group had a significantly higher rate of iatrogenic preterm delivery than the other three groups.

Insulin resistance and oxidative stress, which increase inflammation and endothelial dysfunction, constitute the pathophysiological basis of various metabolic abnormalities [4]. These factors are also the pathophysiological basis of cardiovascular disease, affecting the cardiac structure and hemodynamics. Patients with metabolic disorders may develop reduced LV systolic and diastolic reserve, leading to increased left ventricular stiffness. As a result, left atrial afterload increases [30]. However, the damage of left atrial function caused by metabolic diseases is not only due to hemodynamic changes, but also due to myocardial damage of atrium caused by metabolic disorders. One study showed that in type 2 diabetes, subjects with normal left atrial size and left ventricular filling pressure also had left atrial strain abnormalities, which may indicate that the underlying cause is not of hemodynamic origin [31]. Take atrial fibrillation for instance, the multiple metabolic diseases are the established risk factors for its underlying pathogenesis, namely atrial remodeling, which is the hall mark of impaired atrial function [32]. It is worth noting that endothelial dysfunction may be the link between reduced LA function and poor pregnancy prognosis. Normal vascular endothelial functions include: 1) the exchange of substances between blood and tissues, transportation of nutrients, water and oxygen to organs, and removal of waste molecules and carbon dioxide; 2) the maintenance of normal blood pressure by regulating vasoconstriction and relaxation; and 3) the prevention of blood agglutination and maintenance of a liquid state [33]. In patients with metabolic disorders, endothelial cells are dysfunctional and lose regulatory activity. For example, fetal placental endothelial dysfunction is one of the pathological features of GDM [34] and cannot be rescued by insulin replacement therapy [35]. In patients with preeclampsia, endothelial dysfunction results in increased peripheral resistance, causing a series of maternal obstetrical complications [36]. In addition, endothelial dysfunction persists after delivery, including higher arterial stiffness and a lower reactive hyperemia index value, and is thus associated with subsequent maternal cardiovascular disease [37].

A previous study emphasized the importance of the hemodynamic and morphological aspects of the maternal heart in predicting complications. The authors reported that mid-wall mechanical impairment at 24 weeks’ gestation reflects a decrease in LV diastolic function and predicts adverse pregnancy outcomes [38]^.^ Consistent with the study, Siegmund et al. reported that right ventricular function is altered in pregnant women with coarctation of the aortic valve and is associated with impaired placentation, which is correlated with adverse offspring outcomes [39]. In line with these reports, our study also found that the impairment of the maternal cardiovascular function was associated with adverse outcomes such as iatrogenic preterm delivery.

The limitations of our study should be noted. Firstly, our study did not include postpartum data or the long-term effects of multiple metabolic diseases during pregnancy on maternal. Long-term follow-up study on LA function is needed. Secondly, in the present study, we only compared CMR patients with patients with either PIH or GDM; we did not explore the respective contribution of each metabolic abnormality to reduced LA function. Gestational hypertension and diabetes mellitus are common metabolic abnormalities during pregnancy that raise the concern of clinicians. However, in clinical practice, it is very common to encounter a combination of metabolic abnormalities. In a cohort of 5535 pregnant women, more than 1/4 showed an aggregation of metabolic risk factors [29]. This study emphasizes that LA function in pregnant women with multiple metabolic abnormalities is worse, and this is worthy of attention. Finally, we did not have complete pre-pregnancy data for the participants in this study. However, When we choose our subjects, we excluded patients with hypertension and diabetes before pregnancy.

## Conclusion

In conclusion, we demonstrated in this study that a decrease in the strain during reservoir phase is valuable for predicting adverse outcome in pregnant women with clustering of metabolic risk factors. LASr value ≤ 38.35% might constitute a powerful predictor of iatrogenic preterm delivery.

## Data Availability

The datasets of the current study are not publicly available due to the restrictions by the Beijing Chaoyang Hospital. The authors used this dataset under an agreement with the Beijing Chaoyang Hospital for the present study. The data are available from the corresponding author on reasonable request.

## Abbreviations

CMR: Clustering of metabolic risk factors
PIH: Pregnancy-induced hypertension
GDM: Gestational diabetes mellitus
LASr: Left atrial strain during reservoir phase
pLASRr: Left atrial peak strain rate during reservoir phase
LAScd: Left atrial strain during conduit phase
pLASRcd: Left atrial peak strain rate during conduit phase
LASct: Left atrial strain during contraction phase
pLASRct: Left atrial peak strain rate during contraction phase
LV GLS: Left ventricular global longitudinal systolic strain
LAVi: Left atrial volume index
LA EF: Left atrial ejection fraction
Pre-BMI: Pre-pregnancy body mass index
TG: Triglyceride
HDL-C: High-density lipoproteins-cholesterol
FPG: Fasting plasma glucose
